# Factors associated with skeletal muscle mass in middle‐aged men living with HIV

**DOI:** 10.1002/jcsm.13545

**Published:** 2024-07-17

**Authors:** Yide Xu, Dongdong Wang, Pei Chen, Bufeng Qi, Xiaoting Li, Chunfeng Xie, Jieshu Wu, Lin Li, Gu Gao, Shanshan Geng, Dandan Yang

**Affiliations:** ^1^ Department of Nutrition and Food Safety, School of Public Health Nanjing Medical University Nanjing China; ^2^ Department of Clinical Nutrition, Sir Run Run Hospital Nanjing Medical University Nanjing China; ^3^ Department of Health Management Center, Nanjing First Hospital Nanjing Medical University Nanjing China; ^4^ Department of Sexually Transmitted Diseases and AIDS Center for Disease Control and Prevention of Jiangsu Province Nanjing China

**Keywords:** Acquired immunodeficiency syndrome, Human immunodeficiency virus, Risk factors, Skeletal muscle mass

## Abstract

**Background:**

Despite extensive research on muscle loss in people living with HIV (PLWH), the prevalence and contributing factors specifically among middle‐aged men remain unclear. This study aimed to determine the prevalence of low muscle mass within this demographic and to identify associated factors.

**Methods:**

A total of 378 men living with HIV were enrolled in the study. They were classified into low muscle mass group if they displayed a skeletal muscle index (SMI) <7.00 kg/m^2^ or fell within the lowest quintile of SMI based on the criteria established by the Asian Working Group for Sarcopenia 2019.

**Results:**

Out of the 378 men living with HIV enrolled, 351 had normal muscle mass, while 27 (7.1%) had low muscle mass. Antiretroviral drugs Zidovudine (AZT) (OR = 0.246, *P* = 0.022) and higher serum albumin levels (OR = 0.899, *P* = 0.026) were found to be protective factors against low muscle mass according to quintile grouping. Strong positive associations between SMI and body mass index (BMI), nutritional risk index (NRI), oedema index and fat‐free mass index (FFMI) (*R* > 0.5, *P* < 0.001) were observed. In addition, both BMI (sensitivity = 0.741, specificity = 0.906) and NRI (sensitivity = 0.963, specificity = 0.601) had high sensitivity and specificity in diagnosing low muscle mass, with critical values of 19.85 and 114.177 for BMI and NRI, respectively. The oedema index was the most effective measure of body composition in detecting abnormal fluid retention with high sensitivity (92.6%) and moderate specificity (71.8%) in identifying individuals with low muscle mass. Notably, PLWH with low muscle mass participants had a significantly higher prevalence (92.6%) of a high oedema index compared with those with normal muscle mass (28.2%). This observation indicates that individuals with HIV who experience reduced muscle mass is commonly accompanied with abnormal fluid retention within the body.

**Conclusions:**

Antiretroviral medication types, specifically Zidovudine, BMI and NRI can be independent risk factors for low muscle mass in men with HIV. These factors, along with BMI, could be used conveniently to predict low muscle mass. Furthermore, the association between the oedema index and muscle mass suggests that observing signs of oedema may indicate a risk of low muscle mass in PLWH.

## Introduction

The human immune system is the target of human immunodeficiency virus (HIV), which is primarily transmitted through sex contact, blood, and mother‐to‐child transmission. According to UNAIDS data, 39 million individuals living with HIV were recorded in 2022 worldwide, with 1.3 million new infections and 630 000 deaths caused by AIDS.^1^ In China, there were 1.224 million people living with HIV (PLWH), with 120 000 new cases in 2022.^2^ Currently, AIDS is considered a chronic disease that increases the risk of age‐related conditions including metabolic disorders, muscle loss, frailty, cardiovascular diseases, and osteoporosis.^3^, ^4^

Sarcopenia, characterized by reduced muscle mass and functional decline, is an age‐related condition with poor outcomes.^5^ The initial stage of simple muscle loss is termed ‘presarcopenia’, which precedes the occurrence of sarcopenia.^6^ Studies have shown that muscle loss and central obesity increase the 5‐year all‐cause mortality among people living with HIV.^7^ Although skeletal muscle index (SMI) is frequently used to evaluate muscle loss, relying on bioelectrical impedance analysis (BIA) devices for screening low muscle mass has some limitations.

Muscle metabolism is influenced by HIV, antiretroviral drugs, and nutritional status. In routine nutritional assessment, BMI is a commonly used indicator of body composition.^8^ Studies have demonstrated a correlation between low BMI and low muscle mass, especially in PLWH, as they are more susceptible to muscle mass loss.^9^, ^10^ Similarly, the nutritional risk index (NRI) has been linked to muscle loss in various populations, including the elderly, cancer patients, and individuals with type 2 diabetes,^11^, ^12^, ^13^ suggesting its potential utility as a tool for identifying low muscle mass among people living with HIV. Therefore, measuring BMI and NRI can aid in screening individuals who may have low muscle mass. Detecting abnormalities in these indicators can prompt further assessment of muscle mass, enabling timely interventions to enhance patient quality of life and overall well‐being. Although BIA has not been widely adopted, its role in non‐invasively, inexpensively, and continuously measuring body composition cannot be overlooked.^14^ Among body composition parameters, the oedema index (EI), obtained by dividing extracellular water (ECW) by total body water (TBW), is a reliable marker of fluid volume.^15^ Research indicates that patients with malnutrition have a higher ratio of ECW to TBW when undergoing kidney replacement therapy.^16^, ^17^, ^18^ Furthermore, in individuals with chronic kidney disease and osteosarcopenia not undergoing dialysis, a negative correlation was reported between the oedema index and appendicular skeletal muscle mass index.^19^ Identification of a relationship between a high oedema index and SMI in PLWH with low muscle mass suggests that clinical signs of oedema could potentially indicate low muscle mass, given that a high oedema index often coincides with oedema‐related symptoms.

The present study aimed to investigate the factors related to low muscle mass among men living with HIV in China, including clinical characteristics of HIV infection, antiretroviral drugs, nutritional status and biochemical parameters.

## Methods

### Study participants

This is a hospital‐based cross‐sectional study at Xinkang Hospital of Jiangsu Province, enrolling 378 men living with HIV aged over 18 years. The participants were using antiretroviral therapy for more than 3 months and had a plasma viral load of <200. The exclusion criteria included neurological disorders (e.g., myasthenia gravis, paraplegia, and Parkinson's disease), organ failure (e.g., acute heart failure, renal failure, and respiratory failure), chronic lung disease, and rapid body composition changes within a short period. The study protocol was approved by the Welfare Committee of Nanjing Medical University (2023‐502).

### Demographics and condition of men living with HIV

The interview questionnaire was administered by trained researchers on demographics, virus type, clinical stage, duration of treatment, and comorbidities.

### Anthropometric parameters

The questionnaire collected the following clinical data: height, weight (HW), waist circumference (WC), and hip circumference (HC), along with disease‐related information including medication usage, disease duration, and comorbidities. The waist‐hip ratio (WHR) was calculated by the division of hip circumference (in centimetres) by waist circumference (in centimetres), while body mass index (BMI) was computed by dividing body weight (measured in kilograms) by the square of height (measured in meters). BMI classifications were conducted following the Chinese criteria for adults: which categorized individuals as underweight (<18.5 kg/m^2^), normal weight (18.5–23.9 kg/m^2^), overweight (24–27.9 kg/m^2^), and obesity (≥28 kg/m^2^).

### Biochemical parameters

Several clinical and biochemical measurements were obtained including red blood cell count (10^12^/L), total white blood cell count (10^9^/L), percentage of lymphocytes (%), lymphocyte count (10^9^/L), platelet count (10^9^/L), haemoglobin (g/L), aspartate aminotransferase (AST, U/L), total bilirubin (μmol/L), alanine aminotransferase (ALT, U/L), direct bilirubin (μmol/L), total protein (TP, g/L), alkaline phosphatase (ALP, U/L), albumin (ALB, g/L), urea nitrogen (mmol/L), creatinine (μmol/L), uric acid (μmol/L), total cholesterol (mmol/L), and triglycerides (mmol/L).

The NRI was calculated based on serum albumin level, body weight, and the value of ideal body weight. To obtain the ideal body weight for males, the following formulation was utilized: Ideal body weight (men) = height − 100 − ((height − 150)/4). Computation of the NRI was carried out using the following formula: NRI = (1.519 × serum albumin) (g/L) + 41.7 × (present weight/ideal body weight).

### Body composition measurement

Oedema index, muscle mass, visceral fat grade, fat mass (FM), and fat‐free mass (FFM) were assessed using bioelectrical impedance analysis (BIA) conducted with the TBF‐418 device from TANITA in Tokyo, Japan. This method has high reproducibility and is a suitable alternative to gold standard tests such as magnetic resonance imaging and dual‐energy X‐ray absorptiometry (DXA). The oedema index is the ratio of ECW to TBW, used to assess the distribution of body water. To calculate the skeletal muscle index (SMI), FMI, and FFMI, the sum of limb skeletal muscle mass, FM, and FFM (measured in kilograms) were divided by the square of height (measured in square meters). SMI values ≤7.00 kg/m^2^ for men were classified as low muscle mass, following the criteria established by the Asian Working Group for Sarcopenia (AWGS) in 2019.^20^ Furthermore, participants were divided into quintiles according to their SMI, with individuals in the lowest quintile designated as the low muscle mass group. This categorization was implemented to confirm the findings derived from the AWGS criteria and to address any potential discrepancies stemming from the restricted number of cases identified as having low muscle mass based on the AWGS criteria.

### Statistical analysis

Demographic data, HIV‐related metrics, anthropometric measurements, biochemical parameters, and body composition indicators of men with HIV were assessed using univariate analysis. Continuous variables with a normal distribution were presented as mean and standard deviation, while those with a non‐normal distribution were depicted as median and quartile. Categorical variables were described using frequency and percentage. Group comparisons for categorical variables were performed using either the chi‐square test or Fisher's exact test. Continuous variables were compared using either the Student's *t*‐test or Kruskal–Wallis test. Predictive factors and their association with low skeletal muscle index (SMI) were estimated using multivariable logistic regression, which included only factors with *P* < 0.1 in the univariate analysis. Odds ratios (ORs) and 95% confidence intervals (CIs) were calculated. Spearman rank correlation was conducted to determine the relationship between oedema index, FMI, FFMI, BMI, WC, HC, lymphocyte count, alanine aminotransferase/ALT, and NRI with SMI.

Receiver operating characteristic (ROC) curves were constructed to evaluate the predictive/diagnostic efficacy of different indicators and determine the accuracy of various indicators in diagnosing low muscle mass. Further, to determine the diagnostic performance of various indicators, we calculated the areas under the ROC curve (AUC), sensitivity, specificity, and their respective 95% confidence intervals (CIs). The AUC reflects the overall discriminative capacity of a diagnostic test, with higher values indicating better diagnostic accuracy. To assess the significance of differences between two correlated ROC curves, the DeLong method was employed. This statistical technique allows for robust comparisons, particularly in cases where ROC curves are correlated. Additionally, internal validation of the findings was conducted through bootstrapping with 1000 replicates. Bootstrapping is a resampling method that involves repeatedly drawing samples from the original dataset to estimate the variability of statistical measures and evaluate the robustness of the results. By generating multiple bootstrap samples and analysing their consistency, the internal validity of the study findings can be evaluated, providing confidence regarding the reliability of the observed associations and diagnostic performance metrics.

The R software version 4.2.2, developed by the R Foundation for Statistical Computing, was utilized to conduct all statistical analyses.

## Results

### Demographic and clinical characteristics of men living with HIV

The study enrolled 378 men living with HIV, with 351 having normal muscle mass and 27 with low muscle mass. The participants had a median age of 38.00 [32.00, 45.00] years, predominantly between 30 and 50 years old. The median duration of antiretroviral therapy was 4.44 [2.58, 6.47] years, as shown in Table 1. Moreover, the group with low muscle mass had a lower level of educational attainment (*P*_grouped by AWGS criteria_ = 0.009), a reduced proportion of previous syphilis comorbidity (*P*_grouped by AWGS criteria_ = 0.005) and fewer CD4+ T cells (*P*_grouped by quintile_ = 0.004) (Table 1). Most of the people living with HIV were asymptomatic, and their condition was stable. No significant differences in age, marital status, comorbidities (hypertension, diabetes, and viral hepatitis), virus type, clinical stage, plasma viral load, or medication usage existed between the two groups.

**Table 1:** Demographics and condition of HIV/AIDS

		Grouped by AWGS criteria			Grouped by quintile		
Characteristics	Total (*N* = 378)	Normal muscle mass (*N* = 351)	Low muscle mass (*N* = 27)	*P* value	Normal muscle mass (*N* = 301)	Low muscle mass (*N* = 77)	*P* value
Demographics							
Age (years)	38.00 [32.00, 45.00]	38.00 [32.00, 45.00]	40.00 [33.00, 44.50]	0.520	38.00 [32.00, 44.00]	39.00 [32.00, 47.00]	0.314
Marital status (%)				0.162			
Not married	185 (48.9)	168 (47.9)	17 (63.0)		146(48.5)	39(50.6)	0.799
Married	193 (51.1)	183 (35.7)	10 (40.6)		155(51.5)	38(49.4)	
Education (%)				0.009			0.364
Primary or lower	78 (20.6)	75 (21.4)	3 (11.1)		67 (22.3)	12 (15.6)	
Secondary	221 (58.5)	198 (56.4)	23 (85.2)		171 (56.8)	50 (64.9)	
Tertiary	79 (20.9)	78 (22.2)	1 (3.7)		63 (20.9)	15 (19.5)	
Condition of HIV/AIDS							
Virus type (%)							
HIV.1	378 (100.0)	351 (100.0)	27 (100.0)		301 (100.0)	77 (100.0)	
Clinical stage (%)							
AIDS period (%)	10 (2.6)	9 (2.6)	1 (3.7)	0.528	6 (2.0)	4 (5.2)	0.125
Viral load (copies/mL)	0.00 [0.00, 0.00]	0.00 [0.00, 0.00]	0.00 [0.00, 0.00]	0.857	0.00 [0.00, 0.00]	0.00 [0.00, 0.00]	0.650
CD4**+** T cell count (μL)	351.00 [220.75, 478.75]	355.00 [223.00, 487.00]	281.00 [211.00, 377.50]	0.077	364.00 [229.00, 499.00]	281.00 [199.00, 375.00]	0.004
Duration of treatment (year)	4.44 [2.58, 6.74]	4.44 [2.79, 6.71]	4.72 [1.90, 7.62]	0.880	4.44 [2.87, 6.61]	4.25 [2.31, 7.42]	0.837
Co‐morbidities (%)							
Hypertension (%)				0.493			0.825
No	344 (91.0)	318 (90.6)	26 (96.3)		271 (90.7)	71 (92.2)	
Yes	34 (9.0)	33 (9.4)	1 (3.7)		28 (9.3)	6 (7.8)	
Diabetes (%)				1.000			0.137
No	366 (96.8)	339 (96.6)	27 (100)		289 (96)	77 (100)	
Yes	12 (3.2)	12 (3.4)	0 (0.0)		12 (4.0)	0 (0.0)	
Hepatitis A/B/C (%)				0.733			1
No	342 (90.5)	318 (90.6)	24 (88.9)		272 (90.3)	70(90.9)	
Yes	36 (9.5)	33 (9.4)	3 (11.1)		29 (9.7)	7 (9.1)	
Syphilis (%)				0.005			0.038
No	263 (69.4)	138 (67.6)	25 (92.6)		202 (66.9)	61 (79.2)	
Yes	115 (30.6)	113 (32.4)	2 (7.4)		99 (33.1)	16 (20.8)	
Medication (%)				0.198			0.042
Based on TDF	265 (71.8)	246 (71.9)	19 (70.4)		206 (70.1)	59 (78.7)	
Based on AZT	58 (15.7)	56 (16.4)	2 (7.4)		53 (18.0)	5 (6.7)	
PIs/INSTIs	46 (12.5)	40 (11.7)	6 (22.2)		35 (11.9)	11 (14.7)	

Data are expressed as medians (interquintile ranges) or number (%). The Kruskal–Wallis test was performed for variables that did not satisfy the normality test.

AZT, zidovudine; INSTIs, integrase strand transfer inhibitors; PIs, protease inhibitor; TDF, tenofovir disoproxil.

### Anthropometric, biochemical, and body composition parameters in normal muscle mass and low muscle mass groups

Table 2 presents a summary of anthropometric parameters, biochemical markers, and body composition outcomes across both groups. Following the AWGS classification and quintile stratification, individuals with lower muscle mass among PLWH demonstrated notably lower BMI, WC, and HC compared with those with normal muscle mass. In terms of biochemical indicators, significant distinctions were observed in lymphocyte count, ALT, and AST/ALT ratios between the two groups, while most other parameters did not exhibit significant differences. Moreover, the PLWH with low muscle mass exhibited a significantly low NRI.

**Table 2:** Body composition and biochemical indicators of the participants

		Grouped by AWGS criteria			Grouped by quintile		
Characteristics	Total (*N* = 378)	Normal muscle mass (*N* = 351)	Low muscle mass (*N* = 27)	*P* value	Normal muscle mass (*N* = 301)	Low muscle mass (*N* = 77)	*P* value
Anthropometric parameters							
Body mass index (kg/m^2^)				<0.001			<0.001
Thin	19 (5.0)	10 (2.8)	9 (33.3)		4 (1.3)	15 (19.5)	
Normal	264 (69.8)	246 (70.1)	18 (66.7)		202 (67.1)	62 (80.5)	
Over weight	86 (22.8)	86 (24.5)	0 (0.0)		86 (28.6)	0 (0.0)	
Obese	9 (2.4)	9 (2.6)	0 (0.0)		9 (3.0)	0 (0.0)	
Waist circumference (cm)	85.00 [80.00, 90.00]	85.00 [80.00, 90.00]	82.00 [78.00, 86.00]	0.010	86.00 [81.00, 91.00]	83.00 [78.00, 87.00]	<0.001
Hip circumference (cm)	93.00 [89.25, 97.00]	93.00 [90.00, 97.00]	89.00 [86.50, 92.00]	<0.001	95.00 [91.00, 97.00]	90.00 [87.00, 93.00]	<0.001
Waist‐to‐hip ratio	0.91 [0.88, 0.95]	0.91 [0.88, 0.95]	0.92 [0.88, 0.94]	0.680	0.91 [0.88, 0.95]	0.92 [0.90, 0.95]	0.152
Biochemical parameters							
White blood cell count (10^9^/L)	5.32 [4.48, 6.20]	5.34 [4.48, 6.24]	4.91 [4.39, 6.03]	0.277	5.36 [4.55, 6.29]	4.84 [4.15, 6.01]	0.022
Lymphocyte count (10^9^/L)	1.90 [1.52, 2.41]	1.91 [1.56, 2.44]	1.71 [1.45, 1.97]	0.031	2.00 [1.60, 2.49]	1.71 [1.43, 1.98]	<0.001
Red cell count (10^12/^L)	4.50 [4.07, 4.79]	4.50 [4.06, 4.80]	4.40 [4.09, 4.60]	0.549	4.50 [4.06, 4.79]	4.53 [4.08, 4.80]	0.857
Haemoglobin (g/L)	148.00 [140.00, 156.00]	148.00 [140.00, 156.00]	150.00 [139.50, 156.00]	0.840	148.00 [140.00, 156.00]	147.00 [140.00, 156.00]	0.700
Platelet count (10^9^/L)	213.87 (52.70)	214.13 (52.58)	210.44 (55.20)	0.726	215.03 (53.13)	209.32 (51.07)	0.397
Aspartate aminotransferase/AST(U/L)	24.00 [19.00, 32.00]	24.00 [19.00, 32.50]	21.00 [17.00, 26.00]	0.066	24.00 [19.00, 32.00]	23.00 [18.00, 31.00]	0.545
Alanine aminotransferase/ALT(U/L)	30.00 [20.00, 44.00]	31.00 [20.00, 45.00]	21.00 [14.50, 29.50]	0.008	31.00 [21.00, 46.00]	25.00 [19.00, 37.00]	0.021
AST/ALT	0.84 [0.67, 1.05]	0.83 [0.66, 1.00]	1.08 [0.76, 1.23]	0.012	0.80 [0.65, 1.00]	0.93 [0.75, 1.18]	0.002
Alkaline phosphatase/ALP (U/L)	98.05 [82.98, 118.44]	98.28 [82.59, 119.03]	95.85 [88.06, 111.97]	0.814	96.60 [81.75, 117.81]	104.64 [89.50, 121.55]	0.063
Total bilirubin (μmol/L)	8.16 [6.52, 11.21]	8.17 [6.52, 11.24]	7.84 [6.53, 10.64]	0.741	8.36 [6.58, 11.36]	7.56 [6.45, 10.02]	0.184
Direct bilirubin (μmol/L)	2.05 [1.51, 2.83]	2.05 [1.50, 2.84]	2.11 [1.54, 2.80]	0.787	2.09 [1.50, 2.91]	1.88 [1.53, 2.64]	0.475
Total protein/TP (g/L)	79.10 [75.60, 82.80]	79.20 [75.70, 83.15]	77.20 [74.10, 80.35]	0.102	79.20 [75.80, 83.30]	78.00 [75.00, 81.90]	0.236
Albumin/ALB (g/L)	46.38 (3.70)	46.41 (3.71)	45.94 (3.55)	0.527	46.57 (3.76)	45.65 (3.37)	0.052
Creatinine (μmol/L)	68.69 [61.94, 76.06]	68.17 [61.89, 75.75]	69.42 [66.36, 77.48]	0.328	68.15 [62.04, 75.55]	70.63 [61.89, 77.26]	0.759
Uric acid (μmol/L)	300.28 [256.84, 350.20]	301.32 [258.09, 353.52]	291.24 [249.45, 318.68]	0.220	305.94 [260.45, 356.08]	283.82 [245.79, 319.38]	0.010
Urea nitrogen (mmol/L)	3.84 [3.35, 4.58]	3.84 [3.34, 4.56]	4.13 [3.53, 4.72]	0.280	3.81 [3.29, 4.46]	4.15 [3.51, 4.80]	0.021
Total cholesterol (mmol/L)	3.96 [3.49, 4.48]	3.97 [3.52, 4.48]	3.71 [3.34, 4.18]	0.274	3.98 [3.53, 4.52]	3.81 [3.31, 4.33]	0.118
Triglycerides (mmol/L)	1.54 [1.06, 2.52]	1.56 [1.06, 2.51]	1.43 [1.16, 2.90]	0.903	1.60 [1.10, 2.49]	1.37 [0.90, 2.57]	0.120
NRI	115.45 (7.24)	115.98 (7.10)	108.40 (5.24)	<0.001	116.95 (6.99)	109.60 (5.37)	<0.001
Body composition							
FMI	3.97 [3.18, 5.10]	4.01 [3.23, 5.14]	3.36 [2.56, 4.28]	0.021	4.07 [3.25, 5.19]	3.57 [2.67, 4.34]	0.002
FFMI	18.24 [17.22, 19.20]	18.34 [17.41, 19.25]	15.56 [15.21, 16.24]	<0.001	18.62 [17.80, 19.45]	16.43 [15.70, 16.96]	<0.001
Oedema index	0.39 [0.38, 0.41]	0.39 [0.38, 0.41]	0.41 [0.41, 0.43]	<0.001	0.39 [0.38, 0.40]	0.41 [0.41, 0.43]	<0.001
Evaluation of oedema index; high (%)	124 (32.8)	99 (28.2)	25 (92.6)	<0.001	64 (21.3)	60 (77.9)	<0.001
Evaluation of visceral fat grade; high (%)	108 (28.6)	106 (30.2)	2 (7.4)	0.013	95 (31.6)	13 (16.9)	0.011

Data are expressed as the mean ± standard deviation or number (%). The Kruskal–Wallis U test was performed for variables that did not satisfy the normality test. These variables are expressed as medians (interquartile ranges).

ALT, alanine aminotransferase; AST, aspartate aminotransferase; FFMI, fat free mass index; FMI, fat mass index; NRI, nutritional risk index.

The low muscle mass group also showed significantly reduced FMI and FFMI compared with the normal muscle mass group. Moreover, a higher oedema index was obtained. Moreover, the low muscle mass group had a lower visceral fat grade, but a higher value of oedema index.

### Factors associated with Low skeletal muscle in people living with HIV

After adjusting for correlated variables based univariate analysis, multivariate logistic regression analysis showed that compared with medication based on Tenofovir disoproxil (TDF), the use of Zidovudine (AZT) (OR_grouped by quintile_ = 0.246, *P* = 0.022) associated with a lower risk of low muscle mass, and higher serum albumin levels (OR_grouped by quintile_ = 0.899, *P* = 0.026) and BMI (OR_grouped by quintile_ = 0.423, *P* < 0.001) may be protective factors according to quintile grouping. However, only BMI and syphilis affected the risk of low muscle mass according to the AWGS grouping results (Table 3). Considering the limited sample size, results from quintile grouping are more reliable.

**Table 3:** Factors associated with risk of low muscle mass estimated by multivariate logistic regression analysis (*N* = 378)

	Grouped by AWGS criteria			Grouped by quintile		
Characteristics	OR	95% CI	*P* value	OR	95% CI	*P* value
CD4+ T cell count (μL)	0.999	0.996–1.002	0.441	1	0.998–1.002	0.824
Medication (%)						
Based on TDF	Ref			Ref		
Based on AZT	0.372	0.044–1.917	0.288	0.246	0.067–0.753	0.022
PIs/INSTIs	1.257	0.311–4.546	0.735	0.678	0.235–1.814	0.453
Syphilis (%)						
No	Ref			Ref		
Yes	0.156	0.023–0.608	0.019	0.489	0.222–1.031	0.067
Lymphocyte count	0.734	0.282–1.688	0.496	0.616	0.335–1.075	0.102
Alanine aminotransferase/ALT (U/L)	0.983	0.938–1.007	0.425	1	0.986–1.005	1
AST/ALT	1.186	0.156–6.674	0.859	1.513	0.525–3.988	0.418
Alkaline phosphatase/ALP				1.011	0.999–1.023	0.076
Albumin/ALB				0.899	0.816–0.986	0.026
Uric acid				1.002	0.997–1.006	0.397
Urea nitrogen				1.238	0.927–1.669	0.146
Body mass index	0.42	0.298–0.559	<0.001	0.423	0.331–0.523	<0.001

AZT, zidovudine; INSTIs, integrase strand transfer inhibitors; PIs, protease inhibitor; TDF, tenofovir disoproxil.

Moreover, various anthropometric parameters demonstrated significant correlation with a reduced likelihood of low muscle mass. Notably, higher values of BMI, WC, and HC (OR < 1, *P* < 0.05) were protective against low muscle mass. Similarly, increased FFMI, NRI, and visceral fat grade (OR < 1, *P* < 0.05) were linked to a diminished risk of low muscle mass. Conversely, a higher oedema index (OR_grouped by quintile_ = 14.986, *P* < 0.001) indicated an elevated likelihood of low muscle mass among men living with HIV (Table S1).

### Relationship between SMI and factors influencing Low muscle mass

**Figure 1:**
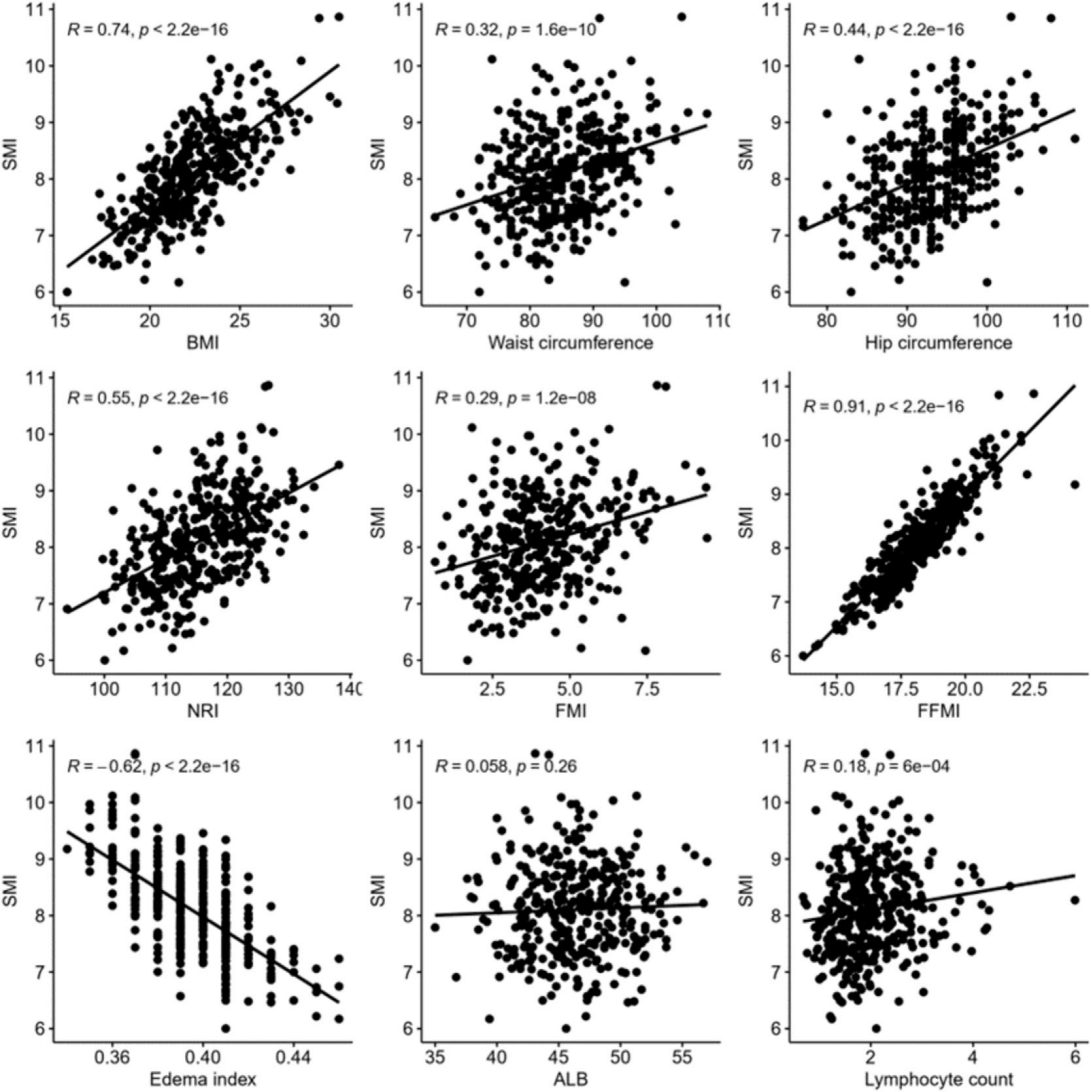
Correlation between various indexes and SMI. ALB, albumin; BMI, body mass index; FFMI, fat free mass index; FMI, fat mass index; NRI, nutritional risk index; SMI, skeletal muscle index.

To investigate the association between low muscle mass and significant factors in the previous logistic regression analysis, correlations were conducted for various variables. BMI (*R* = 0.74, *P* < 0.001), WC (*R* = 0.32, *P* < 0.001), and HC (*R* = 0.44, *P* < 0.001) were significantly correlated with SMI. Notably, BMI revealed a strong positive correlation with SMI (R = 0.74). Among the biochemical parameters, the lymphocyte count and NRI were significantly correlated with SMI, and NRI (*R* = 0.55, *P* < 0.001) showing a particularly strong correlation (Figure 1).

Among body composition parameters, FMI (*R* = 0.29, *P* < 0.001), FFMI (*R* = 0.91, *P* < 0.001), and oedema index (*R* = −0.62, *P* < 0.001) were significantly correlated with SMI, FFMI and oedema index exhibited strong correlations with SMI (Figure 1).

### Evaluation of indicators for diagnosing Low muscle mass in men living with HIV

We analysed the diagnostic efficacy of the aforementioned indicators in identifying low muscle mass in HIV‐positive males using ROC curves and determined the cut‐off values to calculate the sensitivity and specificity of each factor. Our findings revealed that these indicators demonstrated high sensitivity or specificity in predicting low muscle mass among individuals living with HIV. In terms of anthropometric parameters and biochemical parameters, BMI and NRI exhibited high sensitivity and specificity. The cut‐off for BMI was 19.85 and for NRI was 114.177, following the AWGS criteria to define low muscle mass (Table 4). When BMI and NRI were combined, the sensitivity of using BMI or NRI alone was decreased, although there was no significant increase in AUC (*P*_DeLong test, grouped by AWGS criteria_ = 0.232, *P*_DeLong test, grouped by quintiles_ = 0.067) (Table 5). These results showed that these two indicators are potential risk factors for low muscle mass when used in combination, and can predict low muscle mass among people living with HIV.

**Table 4:** Specificity and sensitivity of indicators

	Grouped by AWGS criteria					Grouped by quintile				
Characteristics	AUC	Threshold	Sensitivity	Specificity	95 %CI	AUC	Threshold	Sensitivity	Specificity	95% CI
Body mass index (kg/m^2^)	0.890	19.85	0.741	0.906	0.834–0.945	0.851	21.35	0.779	0.771	0.805–0.897
NRI	0.795	114.177	0.963	0.601	0.731–0.859	0.793	114.663	0.857	0.631	0.743–0.842
BMI + NRI	0.896	0.055	0.926	0.752	0.845–0.947	0.865	0.179	0.844	0.728	0.823–0.907
Waist circumference (cm)	0.649	89.5	0.963	0.302	0.555–0.743	0.632	89.5	0.922	0.336	0.567–0.697
Hip circumference (cm)	0.740	94.5	0.963	0.464	0.657–0.823	0.734	94.5	0.896	0.518	0.677–0.791
FFMI	0.968	16.918	0.963	0.877	0.944–0.991	0.957	17.634	0.948	0.807	0.938–0.976
Oedema index	0.867	0.405	0.926	0.718	0.808–0.926	0.846	0.405	0.779	0.787	0.799–0.893
FFMI + oedema index	0.979	0.147	0.889	0.954	0.960–0.997	0.983	0.23	0.948	0.930	0.973–0.993
FMI1	0.633	3.573	0.593	0.647	0.520–0.746	0.612	4.342	0.753	0.429	0.542–0.682
FMI2	0.633	3.194	0.481	0.758	0.520–0.746					

FMI1 and FMI2 mean that there are two truncation values with the same area under the curve.

AUC, area under curve; BMI, body mass index; CI, credibility interval; FFMI, fat free mass index; FMI, fat mass index; NRI, nutritional risk index.

**Table 5:** Comparative analysis of the discrimination for low muscle mass

		Grouped by AWGS criteria		Grouped by quintile	
	Characteristics	AUC	*P*_DeLong test_	AUC	*P*_DeLong test_
BMI versus BMI + NRI	BMI	0.890	0.232	0.851	0.067
	BMI+NRI	0.896		0.864	
FFMI versus FFMI + EI	FFMI	0.968	0.064	0.957	0.001
	FFMI+EI	0.979		0.983	

AUC, area under curve; BMI, body mass index; EI, oedema index; FFMI, fat free mass index; NRI, nutritional risk index.

**Figure 2:**
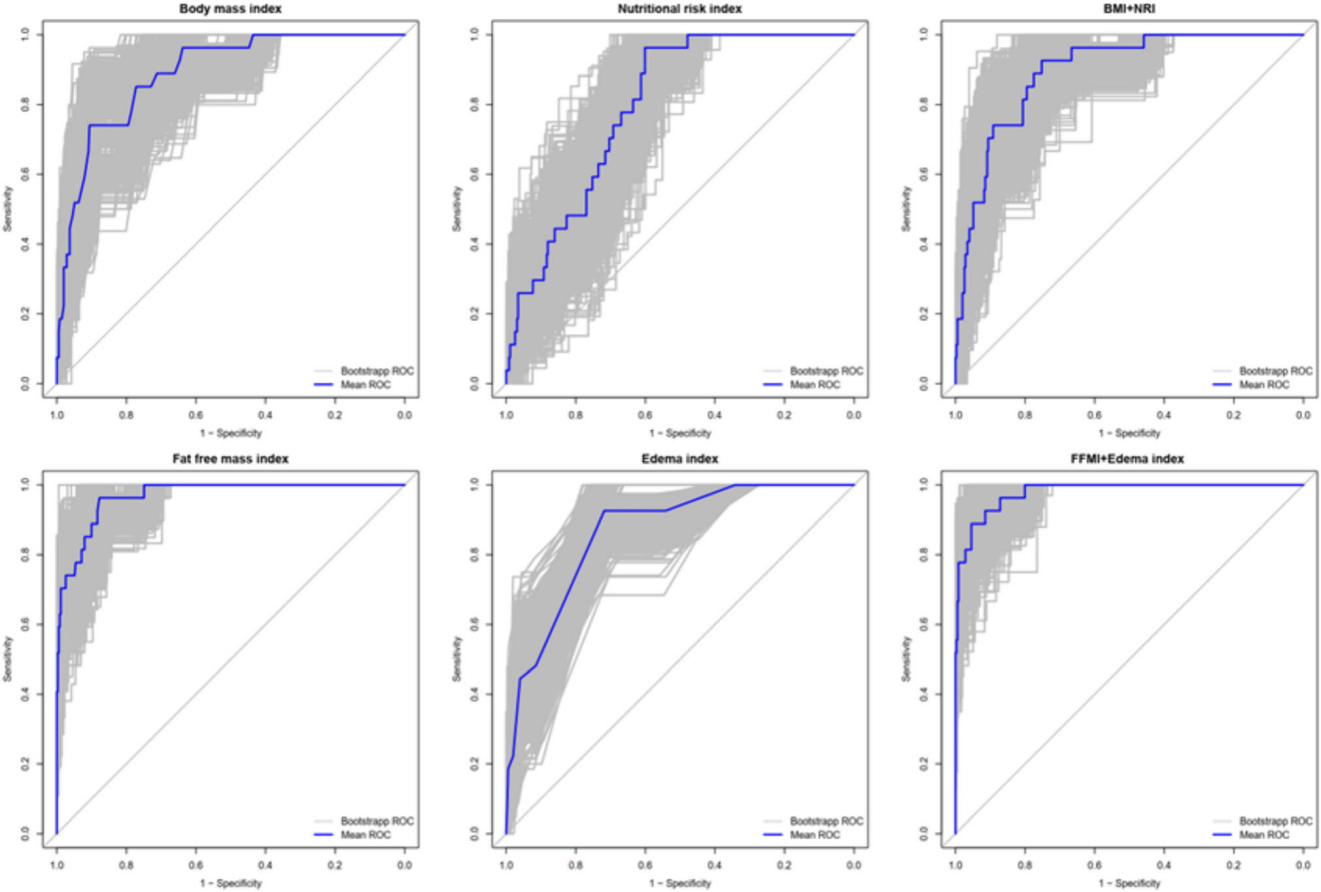
Bootstrap ROC curves of various indexes. BMI, body mass index; FFMI, fat free mass index; NRI, nutritional risk index.

In terms of body composition parameters, following the AWGS standard classification guidelines, both FFMI and the oedema index exhibited high sensitivity and specificity in diagnosing low muscle mass (Table 4). This suggests a potential shared pathophysiological mechanism between oedema and low muscle mass in people living with HIV. Furthermore, we performed 1000 re‐sampling iterations with replacements using Bootstrap to internally validate the reliability of our conclusions (Figure 2). This internal validation process provided further confirmed the robustness of our findings. Similar results were obtained in the analysis grouped by quintile (Table 4, Figure S1).

## Discussion

Our results reveal a 7.1% prevalence of low muscle mass among men with HIV in China, without muscle function loss (normal grip strength test results). This rate is comparatively lower than the low muscle mass and sarcopenia reported in similar studies,^21^, ^22^, ^23^, ^24^, ^25^, ^26^, ^27^ and sarcopenia includes not only muscle mass loss but also a decline in muscle function. Notably, the participants enrolled in this study were middle‐aged men, with a median age of 38 (IQR, 32–45). In contrast, the Korea National Health and Nutrition Examination Survey on a broader population showed that the prevalence of low muscle mass among men aged 40 years and above, with an average age of 57 years, was 3.2%.^28^ Although literature on the prevalence of low muscle mass in normal populations with a mean age of 40 years is limited, it is believed that individuals with HIV may have a much higher prevalence. Our study sheds light on the current state of middle‐aged men living with HIV, with low muscle mass in China.

In the analysis aimed at identifying risk factors for low muscle mass, individuals undergoing AZT medication exhibited a reduced risk compared with those on TDF‐based medication among PLWH, aligning with findings from another study conducted in China.^29^ However, a prior study demonstrated the detrimental effects of AZT on skeletal muscle cells stem from mitochondrial damage, revealing the influence of antiretroviral drugs on muscle mass in PLWH.^30^ In addition to drugs, we found a correlation between low muscle mass and serum albumin levels. This is because HIV infection induces long‐term effects that increase the body's protein consumption and immune activity, causing muscle mass and function decline.^4^ In addition, we investigated the association between inflammatory markers and SMI. However, due to sample size and sampling methods limitations, there was no significant association between immune activation and low muscle mass.

Low BMI and low NRI were significant risk factors for low muscle mass in middle‐aged men with HIV, which is consistent with previous research associating BMI with sarcopenia or muscle mass loss.^31^, ^32^, ^33^ Furthermore, Güç's study showed that GNRI was a risk factor for sarcopenia or low muscle mass in metastatic colorectal cancer patients.^13^ BMI and NRI showed high sensitivity and specificity in diagnosing low muscle mass, whether used alone or in combination, with cut‐off values of 19.85 for BMI and 114.177 for NRI, suggesting they are potentially effective screening tools for early intervention in the absence of body composition measurements in routine clinical practice.

This study presents pioneering evidence for the significant association between the oedema index and SMI in PLWH, showing considerable sensitivity and specificity in detecting low muscle mass. Among individuals with low muscle mass, a significantly larger percentage (92.6%) exhibited a high oedema index compared with those with normal muscle mass (28.2%). Thus, oedema index may be a primary and sensitive marker reflecting alterations in body water distribution.^34^ It can also serve as an indicator of health status, highlighting the need to drink water and nutrition status, particularly among the elderly.^35^ Several studies have observed a correlation between the oedema index and muscle mass. For instance, a Korean survey of non‐dialysis chronic kidney disease patients showed a negative correlation between the oedema index of ND‐CKD patients and their muscle mass index, strength, and physical function.^16^ Similarly, a study investigating rural osteopenia patients reported a negative association between the oedema index and the degree of disability.^19^ Furthermore, an inverse relationship was observed between the oedema index and appendicular muscle mass index (AMMI) in peritoneal dialysis patients.^36^ These studies collectively suggest a certain degree of correlation between the oedema index and muscle mass. This study is the first to reveal a strong negative relationship between the oedema index and low muscle mass in men living with HIV (*R* = −0.62, *P* < 0.001). The high oedema index correlates with clinical signs of oedema. If the oedema index is above the normal range, it may indicate increased water retention in body tissues, which often precipitates signs of oedema.^37^, ^38^ These results suggest that oedema signs can be used to determine whether further assessments are needed to reach a final diagnosis of sarcopenia.

While this study has provided valuable insights, several limitations should be noted. Firstly, it was conducted at a single centre in China among young and middle‐aged men living with HIV, which may limit the generalizability of the findings to a broader population. External validation in women living with HIV and other demographic groups could enhance the reliability of using the oedema index as a screening tool for low muscle mass in people living with HIV. Secondly, due to the limited sample size, this cross‐sectional study only allows for preliminary exploration of the risk factors associated with low muscle mass, rather than establishing causal relationships. Thirdly, our study did not include data on physical activity and diet, which are important confounding factors in the development of muscle loss.

In summary, our results demonstrate that medication type, BMI, and NRI are risk factors for low muscle mass in people living with HIV and can be used for the screening of BMI falls below 19 and NRI below 114. Furthermore, the strong correlation between the oedema index and muscle mass in men living with HIV highlights its potential implications in clinical practice. Signs of oedema are indicated by the high oedema index and thus can predict low muscle mass. Our data can help clinicians to assess low muscle mass in PLWH using BMI, NRI, and oedema signs, thereby initiate appropriate interventions. However, considering the limitations of our study, further investigations, including physical activity and diet data, and enrolling more patients are necessary to overcome these limitations.

## Funding

This research was supported by the National Natural Science Foundation of China (No. 81872607), Connotation Construction Special Fund of Nanjing Medical University, and Connotation Construction Special Fund of Nanjing Medical University's Double First‐Class Initiative.

## Conflict of interest

The authors declare that they have no conflicts of interest.

## Supporting information


**Table S1.** Multivariate logistic regression results of 8 anthropometric parameters or body composition parameters.
**Figure S1.** Bootstrap ROC Curves of various indexes grouped by quartiles. Abbreviations: NRI, Nutritional Risk Index; BMI, body mass index; FFMI, Fat free mass index.

## References

[1] UNAIDS . Global HIV & AIDS statistics — fact sheet. 2023; Available from: https://www.unaids.org/en/resources/fact‐sheet.

[2] National Center for AIDS/STD Control and Prevention, C.C.f.D.C.a.P. In Report on the estimation of HIV/AIDS epidemic in China in 2022; 2022.

[3] Webel AR , Schexnayder J , Cioe PA , Zuñiga JA . A review of chronic comorbidities in adults living with HIV: state of the science. J Assoc Nurses AIDS Care 2021;32:322–346. 33595986 10.1097/JNC.0000000000000240 PMC8815414

[4] Hawkins KL , Brown TT , Margolick JB , Erlandson KM . Geriatric syndromes: new frontiers in HIV and sarcopenia. AIDS 2017;31 Suppl 2:S137–s146. 28471944 10.1097/QAD.0000000000001444 PMC5693390

[5] Cruz‐Jentoft AJ , Bahat G , Bauer J , Boirie Y , Bruyère O , Cederholm T , et al. Sarcopenia: revised European consensus on definition and diagnosis. Age Ageing 2019;48:16–31. 30312372 10.1093/ageing/afy169 PMC6322506

[6] Cruz‐Jentoft AJ , Baeyens JP , Bauer JM , Boirie Y , Cederholm T , Landi F , et al. Sarcopenia: European consensus on definition and diagnosis: report of the European Working Group on Sarcopenia in Older People. Age Ageing 2010;39:412–423. 20392703 10.1093/ageing/afq034 PMC2886201

[7] Scherzer R , Heymsfield SB , Lee D , Powderly WG , Tien PC , Bacchetti P , et al. Decreased limb muscle and increased central adiposity are associated with 5‐year all‐cause mortality in HIV infection. AIDS 2011;25:1405–1414. 21572308 10.1097/QAD.0b013e32834884e6 PMC3933309

[8] Weir CB , Jan A . BMI Classification Percentile And Cut Off Points. In StatPearls. Treasure Island (FL): StatPearls Publishing LLC; 2023, StatPearls Publishing Copyright © 2023. 31082114

[9] Chen F , Xu S , Wang Y , Chen F , Cao L , Liu T , et al. Risk factors for sarcopenia in the elderly with type 2 diabetes mellitus and the effect of metformin. J Diabetes Res 2020. 10.1155/2020/3950404 PMC7563046 33083494

[10] de Almeida LL , Ilha TASH , de Carvalho JAM , Stein C , Caeran G , Comim FV , et al. Sarcopenia and its association with vertebral fractures in people living with HIV. Calcif Tissue Int 2020;107:249–256. 32683475 10.1007/s00223-020-00718-y

[11] Shiroma K , Tanabe H , Takiguchi Y , Yamaguchi M , Sato M , Saito H , et al. A nutritional assessment tool, GNRI, predicts sarcopenia and its components in type 2 diabetes mellitus: a Japanese cross‐sectional study. Front Nutr 2023;10. 10.3389/fnut.2023.1087471 PMC9928854 36819693

[12] Ruan GT , Zhang Q , Zhang X , Tang M , Song MM , Zhang XW , et al. Geriatric nutrition risk index: prognostic factor related to inflammation in elderly patients with cancer cachexia. J Cachexia Sarcopenia Muscle 2021;12:1969–1982. 34585849 10.1002/jcsm.12800 PMC8718015

[13] Güç ZG , Altay C , Özgül HA , Ellidokuz H , Yavuzşen T . GNRI and conut scores: simple predictors of sarcopenia in metastatic colorectal cancer patients. Support Care Cancer 2022;30:7845–7852. 35716261 10.1007/s00520-022-07218-9

[14] Ferguson CE , Lambell KJ . Clinimetrics: bioelectrical impedance analysis in clinical practice. J Physiother 2022;68:280. 35715376 10.1016/j.jphys.2022.05.007

[15] Deurenberg P , Tagliabue A , Schouten FJ . Multi‐frequency impedance for the prediction of extracellular water and total body water. Br J Nutr 1995;73:349–358. 7766559 10.1079/bjn19950038

[16] Kang SH , Kim JC , Cha RH , Han M , An WS , Kim SH , et al. Impact of volume status on sarcopenia in non‐dialysis chronic kidney disease patients. Sci Rep 2022;12:22289. 36566275 10.1038/s41598-022-25135-z PMC9789973

[17] Sukackiene D , Laucyte‐Cibulskiene A , Vickiene A , Rimsevicius L , Miglinas M . Risk stratification for patients awaiting kidney transplantation: role of bioimpedance derived edema index and nutrition status. Clin Nutr 2020;39:2759–2763. 31866127 10.1016/j.clnu.2019.12.001

[18] Kishino K , Enomoto H , Shimono Y , Moriwaki EI , Nishikawa H , Nishimura T , et al. Association of an overhydrated state with the liver fibrosis and prognosis of cirrhotic patients. In Vivo 2020;34:1347–1353. 32354929 10.21873/invivo.11912 PMC7279852

[19] Park SH , Kim MJ , Kim B , Lee GY , Seo YM , Park JY , et al. Association between disability and edema index values in rural older adult osteosarcopenia patients. Yonsei Med J 2022;63:873–880. 36031788 10.3349/ymj.2022.63.9.873 PMC9424777

[20] Ueshima J , Maeda K , Shimizu A , Inoue T , Murotani K , Mori N , et al. Diagnostic accuracy of sarcopenia by ‘possible sarcopenia’ premiered by the Asian Working Group for Sarcopenia 2019 definition. Arch Gerontol Geriatr 2021;97:104484. 34298259 10.1016/j.archger.2021.104484

[21] Almeida TS , Cortez AF , Cruz MRD , Almeida VP . Predictors of sarcopenia in young hospitalized patients living with HIV. Braz J Infect Dis 2021;25:101574. 33861970 10.1016/j.bjid.2021.101574 PMC9392175

[22] Echeverría P , Bonjoch A , Puig J , Estany C , Ornelas A , Clotet B , et al. High prevalence of sarcopenia in HIV‐infected individuals. Biomed Res Int 2018. 10.1155/2018/5074923 PMC6077654 30112397

[23] Luk FWL , Li T , Ho HY , Chan YY , Cheung SK , Wong V , et al. Sarcopenia in people living with HIV in Hong Kong: which definition correlates with health outcomes? J Int AIDS Soc 2022;25 Suppl 4:e25988. 36176015 10.1002/jia2.25988 PMC9522638

[24] Abdul Aziz SA , Mcstea M , Ahmad Bashah NS , Chong ML , Ponnampalavanar S , Syed Omar SF , et al. Assessment of sarcopenia in virally suppressed HIV‐infected Asians receiving treatment. AIDS 2018;32:1025–1034. 29547442 10.1097/QAD.0000000000001798

[25] Wasserman P , Segal‐Maurer S , Rubin DS . High prevalence of low skeletal muscle mass associated with male gender in midlife and older HIV‐infected persons despite CD4 cell reconstitution and viral suppression. J Int Assoc Provid AIDS Care 2014;13:145–152. 24067494 10.1177/2325957413495919

[26] Pinto Neto LF , Sales MC , Scaramussa ES , da Paz CJ , Morelato RL . Human immunodeficiency virus infection and its association with sarcopenia. Braz J Infect Dis 2016;20:99–102. 26626165 10.1016/j.bjid.2015.10.003 PMC9425396

[27] Konishi K , Nakagawa H , Asaoka T , Kasamatsu Y , Goto T , Shirano M . Sarcopenia among people living with HIV and the effect of antiretroviral therapy on body composition. Medicine (Baltimore) 2022;101:e31349. 36281131 10.1097/MD.0000000000031349 PMC9592382

[28] Kim JA , Hwang SY , Yu JH , Roh E , Hong SH , Lee YB , et al. Association of the triglyceride and glucose index with low muscle mass: KNHANES 2008‐2011. Sci Rep 2021;11:450. 33432036 10.1038/s41598-020-80305-1 PMC7801612

[29] Zhang Z , Lin Q , Xu Y , Guan W , Song X , Li Y , et al. Effect of different antiretroviral therapy on muscle mass, bone mineral density, and trabecular bone score in Chinese HIV‐infected males. Arch Osteoporos 2023;18:48. 37041320 10.1007/s11657-023-01238-6

[30] de la Asunción JG , del Olmo ML , Sastre J , Millán A , Pellín A , Pallardó FV , et al. AZT treatment induces molecular and ultrastructural oxidative damage to muscle mitochondria. Prevention by antioxidant vitamins. J Clin Invest 1998;102:4–9. 9649550 10.1172/JCI1418 PMC509058

[31] Merchant RA , Seetharaman S , Au L , Wong MWK , Wong BLL , Tan LF , et al. Relationship of fat mass index and fat free mass index with body mass index and association with function, cognition and sarcopenia in pre‐frail older adults. Front Endocrinol (Lausanne) 2021;12:765415. 35002957 10.3389/fendo.2021.765415 PMC8741276

[32] Xiang T , Fu P , Zhou L . Sarcopenia and osteosarcopenia among patients undergoing hemodialysis. Front Endocrinol (Lausanne) 2023;14. 10.3389/fendo.2023.1181139 PMC10230055 37265691

[33] Tey SL , Chew STH , How CH , Yalawar M , Baggs G , Chow WL , et al. Factors associated with muscle mass in community‐dwelling older people in Singapore: findings from the SHIELD study. PLoS ONE 2019;14:e0223222. 31596873 10.1371/journal.pone.0223222 PMC6785067

[34] Grabosch SM , Shariff OM , Wulff JL , Helm CW . Non‐steroidal anti‐inflammatory agents to induce regression and prevent the progression of cervical intraepithelial neoplasia. Cochrane Database Syst Rev 2014;2014:Cd004121. 24715225 10.1002/14651858.CD004121.pub3 PMC6457632

[35] Lee JE , Jo IY , Lee SM , Kim WJ , Choi HY , Ha SK , et al. Comparison of hydration and nutritional status between young and elderly hemodialysis patients through bioimpedance analysis. Clin Interv Aging 2015;10:1327–1334. 26316728 10.2147/CIA.S86229 PMC4541557

[36] Kang SH , Do JY . Effects of volume status on body composition in incident peritoneal dialysis patients. Eur J Clin Nutr 2020;74:633–641. 32029910 10.1038/s41430-020-0574-y

[37] Bedogni G , Malavolti M , Severi S , Poli M , Mussi C , Fantuzzi AL , et al. Accuracy of an eight‐point tactile‐electrode impedance method in the assessment of total body water. Eur J Clin Nutr 2002;56:1143–1148. 12428182 10.1038/sj.ejcn.1601466

[38] Sartorio A , Malavolti M , Agosti F , Marinone PG , Caiti O , Battistini N , et al. Body water distribution in severe obesity and its assessment from eight‐polar bioelectrical impedance analysis. Eur J Clin Nutr 2005;59:155–160. 15340370 10.1038/sj.ejcn.1602049

