# Supplementary material for: Factors associated with skeletal muscle mass in middle‐aged men living with HIV
Source: J Cachexia Sarcopenia Muscle. 2024 Jul 17;15(5):1965–75. doi: 10.1002/jcsm.13545 (PMC11446698; doi:10.1002/jcsm.13545)
Supplement: Supplementary file 1 — Table S1. Multivariate logistic regression results of 8 anthropometric parameters or body composition parameters. Figure S1. Bootstrap ROC Curves of various indexes grouped by quartiles. Abbreviations: NRI, Nutritional Risk Index; BMI, body mass index; FFMI, Fat free mass index. [file JCSM-15-1965-s001.docx]

**Supplementary table 1 Multivariate logistic regression results of 8 anthropometric parameters or body composition parameters.**

|  | **Grouped by AWGS criteria** | | | **Grouped by quintile** | | |
| --- | --- | --- | --- | --- | --- | --- |
| **Characteristics** | **OR** | **95%CI** | ***P* value** | **OR** | **95%CI** | ***P* value** |
| **Body mass index** | **0.42** | **0.298~0.559** | **<0.001** | **0.423** | **0.331~0.523** | **<0.001** |
| **Waist circumference** | **0.926** | **0.868~0.984** | **0.017** | **0.92** | **0.879~0.96** | **<0.001** |
| **Hip circumference** | **0.848** | **0.774~0.922** | **<0.001** | **0.83** | **0.776~0.882** | **<0.001** |
| **NRI** | **0.854** | **0.788~0.917** | **<0.001** | **0.65** | **0.575~0.723** | **<0.001** |
| **FFMI** | **0.046** | **0.011~0.123** | **<0.001** | **0.049** | **0.019~0.102** | **<0.001** |
| **FMI** | **0.755** | **0.544~1.023** | **0.08** | **0.728** | **0.586~0.893** | **0.003** |
| **Evaluation of edema index** |  |  |  |  |  |  |
| **Normal** | **Ref** |  |  | **Ref** |  |  |
| **High** | **35.854** | **9.173~249.643** | **<0.001** | **14.986** | **7.589~31.42** | **<0.001** |
| **Evaluation of** **visceral fat grade** |  |  |  |  |  |  |
| **Normal** | **Ref** |  |  | **Ref** |  |  |
| **High** | **0.198** | **0.031~0.714** | **0.033** | **0.373** | **0.175~0.746** | **0.007** |

**Grouped by AWGS criteria adjusted by** **CD4+ T cell count, Medication, Syphilis, Lymphocyte count, ALT, AST/ALT; Grouped by quintile adjusted by CD4+ T cell count, Medication, Syphilis, Lymphocyte count, ALT, AST/ALT, Alkaline phosphatase, Albumin, Uric acid, Urea nitrogen.**

**Abbreviations: NRI, Nutritional Risk Index; FMI, Fat mass index; FFMI, Fat free mass index; AST, Aspartate aminotransferase; ALT, Alanine aminotransferase.**


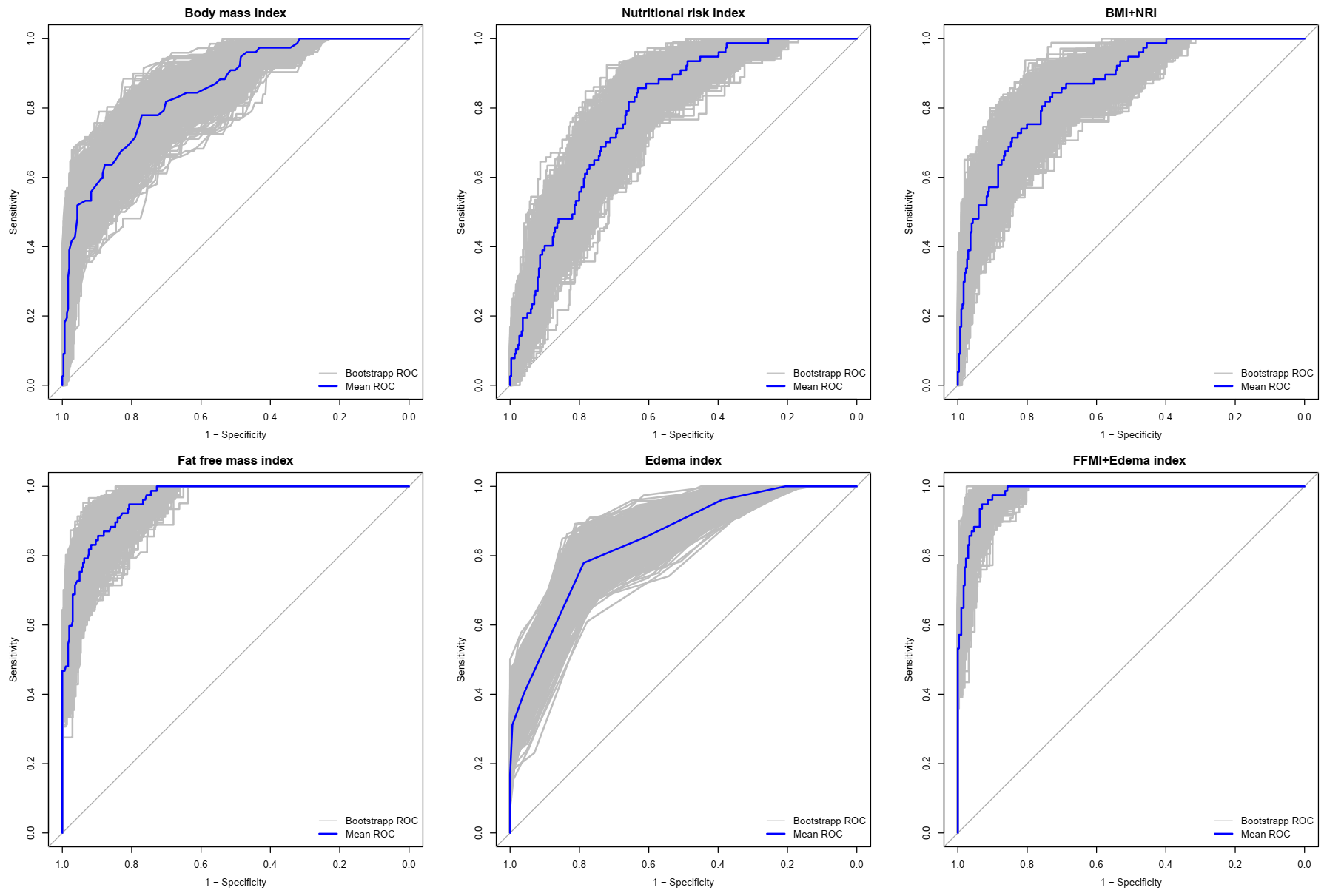


**Supplementary figure 1 Bootstrap ROC Curves of various indexes grouped by quartiles. Abbreviations: NRI, Nutritional Risk Index; BMI, body mass index; FFMI, Fat free mass index.**
